# Myocardial post-conditioning with *Danshen-Gegen *decoction protects against isoproterenol-induced myocardial injury *via *a PKCε/mK_ATP_-mediated pathway in rats

**DOI:** 10.1186/1749-8546-6-7

**Published:** 2011-02-14

**Authors:** Sze Man Wong, Po Yee Chiu, Hoi Yan Leung, Limin Zhou, Zhong Zuo, Philip Y Lam, Kam Ming Ko

**Affiliations:** 1 Division of Life Science, The Hong Kong University of Science and Technology, Hong Kong SAR, China; 2 School of Pharmacy, Faculty of Medicine, The Chinese University of Hong Kong, Hong Kong SAR, China

## Abstract

**Background:**

*Danshen-Gegen *decoction (DG), a Chinese herbal formula, has been demonstrated to be effective for the treatment of coronary heart disease such as myocardial infarction. In the present study, we investigated the effect of DG post-conditioning on isoproterenol (ISO)-induced myocardial injury in rats.

**Methods:**

ISO was injected intraperitoneally (200 mg/kg) to induce acute (2-6 hours) myocardial injury in adult female rats. DG (4 g/kg) was administered *per oral *immediately after the injection of ISO in the rats. Extent of myocardial injury was assessed by measurements of plasma enzyme activities. Myocardial mitochondrial glutathione antioxidant status, lipid peroxidation and mitochondrial calcium ion loading and cytochrome c release were also measured. Effects of inhibitors of protein kinase C-epsilon (PKCε) ranslocation and mitochondrial ATP-sensitive potassium channel (mK_ATP_) on myocardial post-conditioning by DG were investigated.

**Results:**

ISO inflicted acute myocardial injury in the rats as evidenced by increased plasma enzyme activities. DG post-treatment alleviated the ISO-induced acute myocardial injury.

**Conclusion:**

DG post-treatment protected the myocardium against ISO-induced acute injury in rats. The myocardial post-conditioning by DG is likely mediated by PKCε/mK_ATP _signaling pathway.

## Background

Atherosclerosis, which may occur in the coronary artery and is linked to the pathogenesis of coronary heart disease (CHD), involves the deposition of plaque-forming biomolecules (cholesterol and triglycerides in particular) onto the inner wall of arteries. The atherosclerotic coronary artery restricts nutrient and oxygen supply to the myocardium, with resultant ischemia and eventual irreversible tissue damage if the ischemic episode is prolonged with or without reperfusion [1,2].

*Radix Salviae Miltiorrhiza *(*Danshen*) and *Radix Puerariae Lobatae *(*Gegen*) are popular Chinese medicinal herbs used in China, Japan and Korea for the treatment of angina pectoris [3] and myocardial infarction [4,5]. Moreover, *Danshen-Gegen *(DG) decoction has long been used to treat CHD [6]. Previous studies reported that raw *Danshen *and *Gegen *and their isolated compounds produced beneficial effects on cardiovascular function in humans [7], rodents [8] and cultured human endothelial cells [5]. Our recent *ex vivo *study demonstrated that an aqueous extract of DG preconditioned myocardium against ischemia/reperfusion injury in rats [9]. However, whether the DG extract can exert any direct beneficial effect on the myocardium immediately after ischemic or oxidative challenge remains to be investigated. The cardioprotection by ischemic post-conditioning is likely linked to the activation of an adenosine-mediated reperfusion-injury salvage kinase (RISK) pathway [10] and a tumor necrosis factor-α-mediated survivor activating factor enhancement (SAFE) pathway [11]; both signaling pathways may target mitochondria via the activation of protein kinase C-epsilon (PKCε), thereby opening a mitochondrial ATP-dependent potassium channel (mK_ATP_), leading to inhibition of a mitochondrial permeability transition and ultimately cardioprotection [12-16].

Isoproterenol [ISO, 1-(3,4-dihydroxyphenyl)-2-isopropylaminoethanol hydrochloride (7)] is a synthetic catecholamine and a potent β_1_/β_2_-adrenergic receptor agonist [17]. A single administration of ISO at large doses or multiple administrations at lower doses could induce myocardial infarction, presumably due to the generation of reactive oxygen species (ROS) through auto-oxidation [18]. ISO-induced myocardial necrosis was associated with alterations in membrane permeability and the subsequent disruption of structural and functional integrity of myocardial membranes [19]. ISO-induced pathophysiological and morphologic alterations in rat hearts resembled clinical manifestations of myocardial infarction in humans [10,20,21].

The present study investigates the effects of myocardial post-conditioning by DG in a rat model of ISO-induced acute myocardial injury. Inhibitors of PKCε translocation and mK_ATP _were used to study the underlying mechanism(s) of myocardial post-conditioning-induced by DG treatment.

## Methods

### Materials

*Radix Salviae Miltiorrhiza *and *Radix Puerariae Lobatae *were purchased from Si Chuan Zhong Jiang Xiang (Sichuen and Yang Jiang, Gaungdong, China) respectively and authenticated by an herbalist working for the Institute of Chinese Medicine (ICM) at The Chinese University of Hong Kong by morphological characterizations and thin layer chromatography in accordance with the Chinese Pharmacopoeia [22]. Voucher specimens of *Radix Salviae Miltiorrhiza *(#2008-3088b) and *Radix Puerariae Lobatae *(#2008-3167b) were deposited in the ICM. DG extract (*Danshen *and *Gegen*, 7:3, w/w) of an optimized ratio as assessed by cardioprotection against ischemia/reperfusion injury [9] was prepared in large-scale for experimental and clinical investigations. Herbs were soaked in water (1:10, w/v) for 75 min, followed by extraction in boiling water for 60 min. The extraction procedure was repeated twice with boiling water (1:8) for 60 min and 30 min. The pooled aqueous extracts were concentrated under reduced pressure at 60°C and the concentrate was spray-dried to obtain the powdered form of DG extract with a yield of 10.1%.

### Chemical analysis of the DG extract

Major components in the DG extract were identified and quantified according to our previous study with minor modifications in terms of instrument and chromatographic conditions [23]. Briefly, a Waters high performance liquid chromatography (HPLC) system (Waters, USA) equipped with a 2695 solvent delivery module and a 996 photodiode UV detector was used. The chromatographic separation of the analytes was achieved by an Agilent Eclipse XDB-C_18 _column (5250 × 4.6 mm; 5 μm particle size, Agilent Technologies, USA) connected to an Agilent C_18 _guard column (Agilent Technologies, USA). The mobile phase consisting of 0.5% acetic acid in acetonitrile (solvent A) and 0.5% acetic acid in water (solvent B) was run with gradient elution at a flow rate of 1 mL/min. The linear gradient elution was carried out as follows: solvent A was kept at 5% for the first 5 min and increased to 10%, 17%, 35% and 90% in the next 13 min, 12 min, 10 min and 3 min respectively; it was then returned to 5% in 5 min and equilibrated for 15 min before the next injection. HPLC analysis indicated that the DG extract contained the following marker compounds (μg/100 mg; mean ± SD, *n *= 3): *danshensu *(1868.2 ± 33.7), salvianolic acid B (1345.7 ± 18.5), protocatechuic aldehyde (78.3 ± 3.9), puerarin (1760.1 ± 23.4), daidzein 8-C-apiosyl-glucoside (404.1 ± 8.1), daidzin (159.4 ± 3.3) and daidzein (162.9 ± 1.4). Pharmacokinetics studies indicated that only danshensu, puerarin and daidzein were detectable in plasma at 30 min after oral administration of DG extract to rats at a dose of 0.15 g/kg (unpublished data).

### Animals

Adult female Sprague-Dawley rats (8-10 weeks; 175-225 g) were housed in an air/humidity-controlled room with 12-hour dark-light cycle at approximately 22°C and allowed food and water *ad libitum *in the Animal and Plant Care Facility of the Hong Kong University of Science and Technology (HKUST) throughout the experiments. All experimental procedures were approved by the Research Practice Committee at the HKUST.

### Induction of acute myocardial injury

Animals were randomly assigned to various groups of six animals in each for the induction of myocardial injury with or without post-treatment with the DG extract. Animals received an intraperitoneal (ip) injection of ISO (Sigma-Aldrich, USA) at a single dose of 200 mg/kg for the induction myocardial injury [24]. Preliminary studies indicated that the ISO administration increased plasma enzyme activities within six hours in the rats. Control animals received the vehicle (saline) only. Blood samples were obtained from phenobarbital-anesthetized (120 mg/kg, ip) rats at increasing time intervals (2, 4 and 6 hours) post-ISO administration. These rats were then sacrificed by cardiac excision. Myocardial ventricular tissue samples were obtained for the preparation of cytosolic and mitochondrial fractions for biochemical analyses. Basal values of plasma enzyme activities and myocardial mitochondrial parameters were obtained from animals sacrificed immediately after the injection of saline.

### DG post-treatment protocol

Animals were intragastrically administered with the DG extract at a dose of 4 g/kg immediately after intraperitoneal injection of ISO in the rat model of ISO-induced acute myocardial injury. Preliminary studies indicated that oral administration of the DG extract at 2 g/kg did not produce any detectable changes in plasma enzyme activities four hours after intraperitoneal injection of ISO in rats.

### Inhibitors of PKCε and mK_ATP_

PKCε translocation inhibitor (Calbiochem, Germany, CAT# 539522) and 5-hydroxydecanoate (5-HD) (Sigma-Aldrich Chemical, USA; CAT# H135), which are inhibitors of PKCε and mK_ATP _respectively, were dissolved in DMSO at a concentration of 400 μg/mL. Rats were injected (ip) with the inhibitor(s) at 400 μg per kg of body weight for one hour prior to the intragastric administration of DG extract or vehicle. Control animals received 1.6% DMSO in saline.

### Preparation of plasma samples and myocardial mitochondrial/cytosolic fractions

Blood was drawn from phenobarbital-anesthetized rats by cardiac puncture into a syringe rinsed with 5% Na_2_EDTA as anti-coagulant (10%, v/v). The blood sample was centrifuged (Himac CF 9RX, Hitachi Koki Co., Ltd., Japan) at 600 × *g *for 10 min at 4°C. The supernatants were collected as plasma samples.

Myocardial ventricular tissue samples were rinsed with ice-cold isotonic buffer (210 mM mannitol, 70 mM sucrose, 5 mM HEPES, 1 mM EGTA, pH7.4, 0.2 mg/mL soybean trypsin inhibitor, 0.2 mg/mL bacitracine, 0.16 mg/mL benzamidine). Tissue homogenates were prepared by homogenizing 0.6 g of minced tissue in 6 mL ice-cold isotonic buffer in a Teflon-in glass homogenizer (Glas-Col, USA) at a speed of 1600 rpm for 20 strokes on ice. The homogenates were centrifuged (Himac CF 9RX, Hitachi Koki Co., Ltd., Japan) at 600 × *g *for 20 min at 4°C. Pellets collected from the supernatant were resuspended with the same volume of ice-cold homogenizing buffer (but without the protease inhibitors) and re-centrifuged (Himac CF 9RX, Hitachi Koki Co., Ltd., Japan) at 600 × *g*. The procedure was repeated twice. After pooled supernatants (4 volumes total) were centrifuged (Himac CR21G, Hitachi Koki Co., Ltd., Japan) at 9200 × *g *for 30 min, the mitochondrial pellets were collected. The supernatants were saved for the preparation of cytosolic fractions. The mitochondrial pellets were then washed with the same volume of ice-cold sucrose buffer (210 mM mannitol, 70 mM sucrose, 5 mM HEPES-KOH; pH7.4) and the mixtures were centrifuged at 9,200 × *g *for 30 min. The washing procedure was repeated once. The mitochondrial pellets were resuspended in 1.0 mL of ice-cold sucrose buffer and constituted the mitochondrial fractions. Cytosolic fraction was prepared from the above supernatant was centrifuged (Optima TLX Ultracentrifuge 120, Beckman Coulter Inc., USA) at 100,000 × *g *for 60 min at 4°C.

### Biochemical analysis

Lactate dehydrogenase (LDH) activity in plasma sample was measured as described by Vanderlinde [25]. Plasma aspartate aminotransferase (AST) activity was measured with an assay kit (Sigma-Aldrich Chemical, USA). An aliquot (180 μL) of reconstituted AST assay solution was mixed with 20 μL plasma sample in a 96-well micro-titer plate. Absorbance changes of the reaction mixture in a final volume of 200 μL were monitored with a Victor ^3 ^Multi-Label Counter (Perkin-Elmer, USA) at 340 nm for 5 min at 37°C. Plasma creatine phosphokinase (CPK) activity was measured with an assay (Sigma-Aldrich Chemical, USA). An aliquot (200 μL) of reconstituted CPK assay solution was mixed with 5 μL plasma sample in a 96-well micro-titer plate. Absorbance changes of the reaction were monitored with a Victor^3 ^Multi-Label Counter (Perkin-Elmer, USA) at 340 nm for 5 min at 37°C. Aliquots (210 μL) of mitochondrial fractions were measured for reduced glutathione (GSH) according to a method by Griffith [26]. Aliquots (250 μL) of mitochondrial fractions were measured for the malondialdehyde (MDA) level, an indirect index of lipid peroxidation according to an HPLC method by Cheng *et al. *[27]. Mitochondrial glutathione reductase (GRD) and Se-glutathione peroxidise (GPX) activities were measured as described by Chiu *et al. *[28]. Mitochondrial isocitrate dehydrogenase (ICDH) activity was measured according to the method by Popova *et al. *[29]. Plasma and mitochondrial parameters were expressed as the percentage of control (*ie *basal value in saline injected animals). Basal values of plasma and mitochondrial parameters were shown in Table 1. Time-dependent changes in plasma enzyme activities and mitochondrial antioxidant components as well as MDA production were quantified according to the area under/or above the curve. Effects of DG post-treatment on ISO-induced changes were expressed in percentage (%) of protection in relation to the corresponding data obtained from DG-untreated animals.

**Table 1:** Basal values of plasma enzyme activities and myocardial mitochondrial antioxidant parameters in rats

	LDH	AST	CPK	GSH	GR	GPX	ICDH	MDA
	U/L			nmol/mg protein	mU/mg protein			pmol/mg protein
Mean (SD) (*n *= 6)	129.8 (10.8)	31.2 (2.68)	158.1 (20.4)	4.3 (0.25)	2.4 (0.24)	2.8 (0.26)	308.8 (23.0)	90.5 (5.39)

Plasma lactate dedydrogenase (LDH), aspartate aminotransferase (AST) and creatine phosphokinase (CPK) activities, as well as myocardial mitochondrial reduced glutathione (GSH) level and glutathione reductase (GR), Se-glutathione peroxidase (GPX), and isocitrate dehydrogenase (ICDH) activities, and malodialdehyde (MDA) level were measured in rats immediately after an intraperitoneal injection of saline.

Mitochondrial Ca^2+ ^content was determined by a Ca^2+^-sensitive fluorescence probe Fluo-5N AM ester (Molecular Probe, USA) on a Victor ^3 ^Multi-Label Counter (Perkin-Elmer, USA) [30]. The Ca^2+ ^dissociation constant (K_d_) was determined by a Ca^2+ ^calibration kit (Molecular Probe, USA) in a range of 1-1000 μM, with an estimated K_d _value of 98 μM, which was in good agreement with the data provided by the manufacturer. An aliquot (25 μL) of mitochondrial fraction (0.5 mg/mL final concentration) was mixed with 25 μL of incubation buffer (100 mM KCl and 30 mM MOPS; pH7.2) in 96-well black micro-titer plate. The mixture was incubated at 25°C for 15 min and then 25 μL digitonin (50 μg/mL) and 25 μL Fluo-5N AM ester (1 μM in 0.005% Pluronic F-127) were added to the mixture. This reaction mixture was incubated at 25°C for 30 min; the fluorescence was measured at 488 nm (excitation) at 532 nm (emission). The mitochondrial Ca^2+ ^content was estimated with a standard calibration curve and presented in μmol/mg of protein.

Mitochondrial cytochrome c release was indirectly assessed by the measurement of cytosolic cytochrome c levels using Western blot analysis [31]. Total cytosolic fractions with equal amounts of protein (40 μg of protein) were subjected to 15% SDS-PAGE, followed by immunoblotting using specific antibodies of cytochrome c (clone 7H8.2C12, BD PharMingen, USA). The extent of mitochondrial contamination in the cytosolic fractions, which was determined using specific antibodies against complex IV and complex IV protein band, was undetectable in cytosolic fractions (data not shown). The protein-blot analysis was performed with an ECL Western Blotting System (Cell Signaling Technology, USA) and the protein bands were quantified by densitometry. The cytochrome c release was estimated from the amount (arbitrary units) of cytochrome c normalized with reference to actin (1:5000, Sigma Chemical, USA) levels (arbitrary units) in the sample.

### Protein assay

Protein concentration was determined with a Bio-Rad protein assay kit (USA). An aliquot (10 μL) of diluted mitochondrial or cytosolic sample was added to the wells of a 96-well micro-titer plate; then 200 μL of 5-fold diluted Bio-Rad assay reagent was added. The mixture was stood at room temperature for 5 min. Absorbance of the mixture was measured at 570 nm. Protein concentration was determined with a calibration curve using bovine serum albumin as standard.

### Statistical analysis

Data were analyzed by one-way ANOVA. *Post-hoc *tests for pair-wise multiple comparisons were done with Least Significant Difference test with SPSS statistical software (SPSS, USA). Comparisons between two groups were performed with Student's t test. Statistical significance was determined at *P *value < 0.05.

## Results

### Effects of DG post-treatment on plasma enzyme activities in ISO-challenged rats

**Figure 1:**
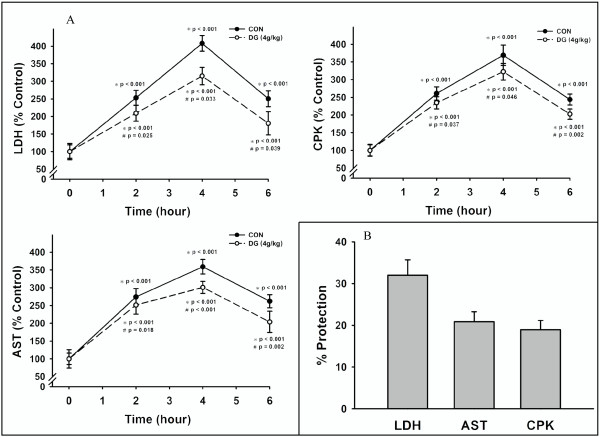
**Effects of DG-post-treatment on plasma enzyme activities in ISO-challenged rats**. Animals were administered intraperitoneally with isoproterenol (ISO) at a dose of 200 mg/kg. Control animals received an injection of saline. DG extract was administered *per oral *at a dose of 4 g/kg immediately after the ISO challenge. Animals were sacrificed at increasing time intervals (2, 4, 6 hours) after ISO challenge. (A) Plasma lactate dehydrogenase (LDH), asparate aminotransferases (AST) and creatine phosphokinase (CPK) activities were measured. (B) The degree of protection against ISO-induced increases in plasma enzyme activities in DG-treated animals was estimated as described in Methods. Values are means ± SD (*n *= 6). * Significantly different from animals receiving saline injection without ISO; ^# ^significantly different from the time-matched ISO-challenged animals without DG post-treatment

As shown in Figure 1a, ISO treatment caused time-dependent increases in plasma enzyme activities, indicative of myocardial injury, with the maximal stimulation at four hours post-ISO challenge. At six hours after post-ISO challenge, the plasma enzyme activities were still significantly higher (144-162%; *P *< 0.001) than the basal values of animals receiving only saline injection. DG treatment (4.0 g/kg) immediately after the ISO challenge decreased the extent of increases in plasma enzyme activities. From the time-dependent changes in plasma enzyme activities as quantified by the area under the curve (AUC), we found that DG post-treatment protected against the ISO-induced increases in plasma enzyme activities by 32% (LDH; *P *= 0.033), 21% (AST; *P *< 0.001) and 19% (CPK; *P = *0.046) (Figure 1b).

### Effects of DG post-treatment on mitochondrial glutathione antioxidant status and lipid peroxidation in ISO-challenged rat hearts

**Figure 2:**
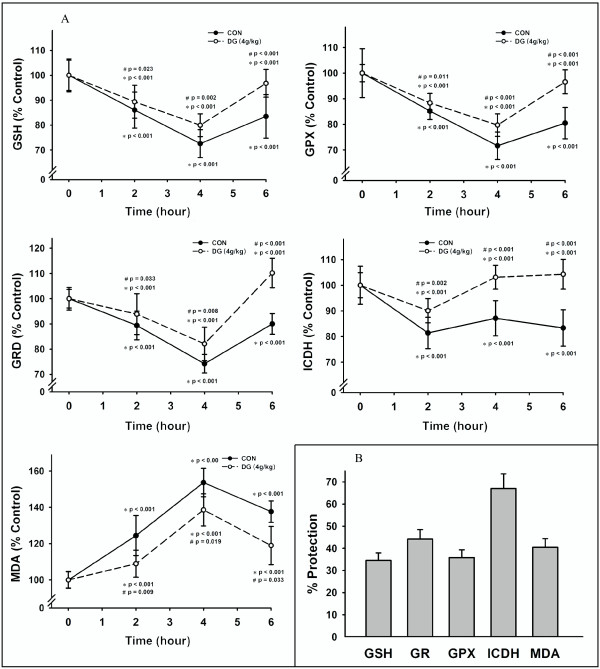
**Effects of DG post-treatment on mitochondrial glutathione status and lipid peroxidation in ISO-challenged rat hearts**. (A) Mitochondrial reduced glutathione (GSH) level, glutathione reductase (GR), Se-glutathione peroxidase (GPX) and isocitrate dehydrogenase (ICDH) activities as well as malondialdehyde (MDA) level were measured. (B) The degree of protection against ISO-induced changes in mitochondrial parameters was estimated as described in Methods. Values are means ± SD (*n *= 6). * Significantly different from animals receiving saline injection without ISO; ^# ^significantly different from the time-matched ISO-challenged animals without DG post-treatment

The ISO-induced myocardial injury was associated with an impairment in myocardial mitochondrial antioxidant status in rats, as evidenced by the time-dependent and biphasic changes in GSH level as well as GRD and GPX activities, with the maximal degree of inhibition 26-28%; *P *< 0.001) at four hours after post-ISO challenge (Figure 2a). The mitochondrial ICDH activity was also suppressed but showed an early recovery two hours after the ISO challenge. The ISO-induced impairment in mitochondrial glutathione antioxidant status was paralleled by an increased extent of mitochondrial lipid peroxidation in rat hearts, as indicated by the time-dependent increase in MDA production, with the maximal stimulation (54%; *P *< 0.001) at four hours after ISO challenge. The protection against ISO-induced myocardial injury afforded by DG post-treatment was associated with the improvement in myocardial mitochondrial glutathione antioxidant status, as assessed by GSH level (35%; *P *= 0.002) (% protection with respect to non-DG-treated and ISO-challenged rats), GRD (45%; *P *= 0.008), GPX (36%; *P *< 0.001) and ICDH (68%; *P *< 0.001) activities as well as the suppression of mitochondrial lipid peroxidation (41%; *P = *0.019) (Figure 2b).

### Effects of DG post-treatment on mitochondrial Ca^2+ ^loading and cytochrome c release in ISO-challenged rats

**Figure 3:**
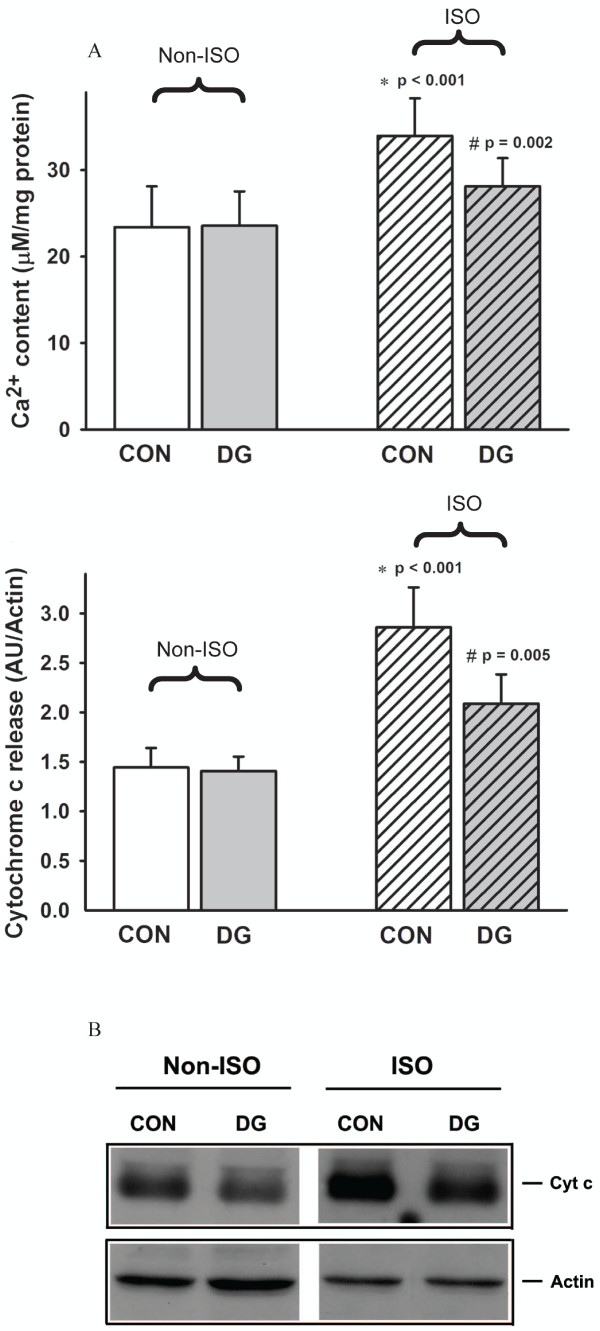
**Effects of DG post-treatment on mitochondrial Ca^2+ ^loading and cytochrome c release in ISO-challenged rat hearts**. Animals were sacrificed at four hours after ISO challenge. Myocardial mitochondrial Ca^2+ ^content and cytochrome c release were measured. The lowest panel shows the representative immuno-stained band of cytochrome c of myocardial cytosolic fractions prepared from various experimental groups. The non-striped bar represents the non-ISO challenged group and the striped bar represents the ISO-challenged group. Values are means ± SD (*n *= 6). * Significantly different from the non-ISO-challenged animals without DG treatment (*ie *CON); ^† ^significantly different from the ISO-challenged CON

ISO challenge increased mitochondrial Ca^2+ ^content (45%; *P *< 0.001) and cytochrome c release (98%; *P *< 0.001) at four hours after ISO challenge in rat hearts (Figure 3). While DG treatment did not affect mitochondrial Ca^2+ ^content and cytochrome c release, it significantly decreased the extent of ISO-induced increases in mitochondrial Ca^2+ ^level and cytochrome c release, with the degree of protection at 56% (*P *= 0.002) and 52% (*P *= 0.005) respectively.

### Effects of PKCε and mK_ATP _inhibitors on myocardial protection by DG post-treatment

**Figure 4:**
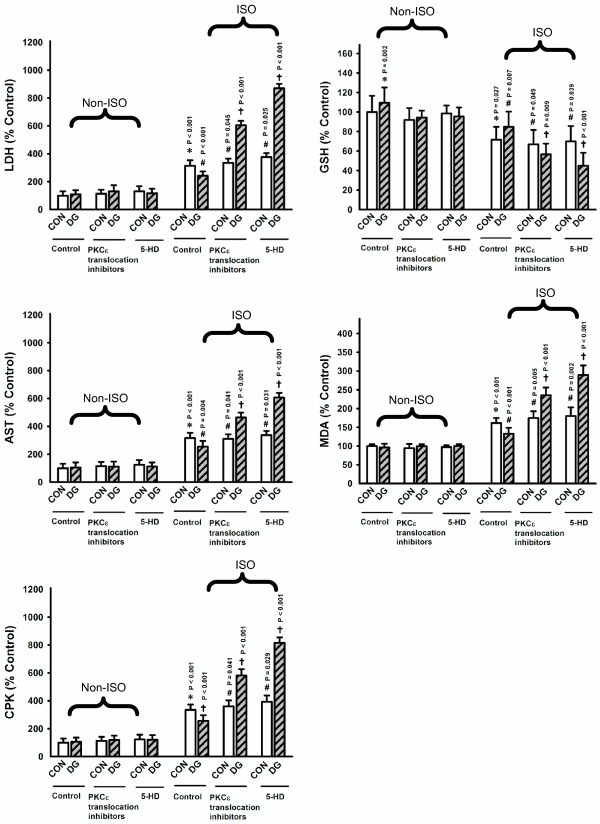
**Effects PKCε and mK_ATP _inhibitors on myocardial protection afforded by DG post-treatment**. Animals were sacrificed at four hours after ISO challenge. PKCε translocation inhibitor and mK_ATP _blocker (5-hydroxydecanoate, 5-HD) were intraperitoneally administered at a dose of 400 μg/kg one hour prior to the administration of the DG extract. Plasma enzyme activities and myocardial mitochondrial antioxidant parameters were measured as described in Figures 1 and 2. The non-striped bar represents the non-ISO-challenged group and the striped bar represents the ISO-challenged group. Values are means ± SD (*n *= 6). * Significantly different from the non-ISO-challenged CON; ^# ^significantly different from the ISO-challenged CON with inhibitors; ^† ^significantly different from the respective ISO-challenged CON

To investigate the signaling pathway involved in the DG-induced myocardial protection, we examined the effects of PKCε and mK_ATP _on myocardial protection against ISO-induced injury by DG post-treatment in rats (Figure 4). The ISO-induced myocardial injury was assessed at four hours after ISO challenge. While the treatment with PKCε translocation inhibitor (400 μg/kg, ip) did not affect the ISO-induced myocardial injury, it completely abrogated the cardioprotection by DG post-treatment, with the degree of myocardial injury slightly higher than that of DG-untreated and ISO-challenged animals. The administration of mK_ATP _blocker (5-HD, 400 μg/kg, ip) also did not affect the ISO-induced myocardial injury but completely abolished the DG-induced cardioprotection against ISO challenge, with a much higher extent of myocardial injury than that of DG-untreated and ISO-challenged rats.

## Discussion

As the pathological changes of myocardial injury caused by acute or multiple ISO treatment resemble the clinical manifestations of myocardial infarction [10,20,21], *eg *the ISO-induced necrotic cells' leakage of housekeeping enzymes such as LDH, AST and CPK from the myocardium to blood, the measurement of these enzyme activities is a reliable assessment for the extent of ISO-induced myocardial injury. Our results showed that ISO administration inflicted acute myocardial injury in rats and that DG treatment immediately after the ISO challenge protected the myocardium against such injury. Preliminary studies indicated that histological changes such as fragmentation of muscle fibers and leukocyte infiltration were not observable in apical ventricular tissue at four hours after ISO challenge in rats. Thus, we did not include histopathological analysis in the present study; however, another study indicated that DG treatment at 24 hour after ISO challenge also protected against myocardial damage in rats, as assessed by plasma enzyme activities and histological parameters (unpublished data). The development of ISO-induced myocardial injury involves ROS-mediated processes [32]. Consistent with this, the ISO-induced myocardial injury was accompanied by the impairment in mitochondrial glutathione antioxidant status and the enhancement in mitochondrial lipid peroxidation in rat hearts. Post-treatment with the DG extract partially reversed the altered myocardial mitochondrial antioxidant parameters in ISO-challenged rats.

Impairment in mitochondrial glutathione antioxidant status renders the cardiomyocytes more susceptible to oxidative stress [33]. The imbalance between ROS generation and glutathione redox cycling may lead to increased mitochondrial Ca^2+ ^loading, which eventually leads to a mitochondrial permeability transition (MPT). The opening of MPT pores is triggered by stimuli such as oxidants, high mitochondrial Ca^2+ ^content and/or depletion of adenine nucleotides [34]. MPT decreases mitochondrial ATP synthesis and causes cytochrome c release from the mitochondrial inner membrane, resulting in necrotic and/or apoptotic cell death [35]. In the rat model of ISO-induced myocardial injury, DG post-treatment may inhibit mitochondrial Ca^2+ ^uptake (as indicated by the decrease in mitochondrial Ca^2+ ^level) and prevent the onset of MPT (as indicated by the decrease in mitochondrial cytochrome c release), thereby protecting against ISO-induced myocardial injury. The ability of DG post-treatment to inhibit MPT may be related to the enhancement in mitochondrial glutathione antioxidant status [36]. While GPX suppresses the oxidation of mitochondrial membrane lipids by removing organic hydroperoxides generated from ROS-mediated reactions [37], glutathione redox cycling, which involves the GR- and ICDH-catalyzed reactions in GSH regeneration and NAPDH production respectively, can sustain the mitochondrial GSH level under oxidative stress conditions [38].

The cardioprotection against ISO-induced injury by DG post-treatment was abrogated by PKCε or mK_ATP _inhibition, suggesting the involvement of PKCε activation and mK_ATP _opening in the process of myocardial post-conditioning by DG. PKCε is a member of a novel group of the PKC family of serine and threonine kinases that are involved in a wide range of physiological processes including mitogenesis, cell survival under stressful conditions, metastasis and transcriptional regulation [39]. It has been postulated that the activation of RISK and SAFE pathways involved in myocardial ischemic post-conditioning might activate PKCε and mK_ATP_, thereby inhibiting the MPT [12-16]. The aggravation of ISO-induced myocardial injury by DG treatment in the presence of PKCε translocation inhibitor may be related to the pro-oxidant action of DG. Moreover, the activation of signal transducers and activators of transcription protein-3 (STAT-3) through the SAFE pathway increased the transcription of antioxidant genes such as those for γ-glutamyl cysteine ligase (for GSH synthesis), GRD and GPX [40-42] which are major determinants of cellular/mitochondrial glutathione antioxidant status. While the mitochondrial glutathione antioxidant status was enhanced by DG post-treatment in ISO-challenged rat hearts, our preliminary studies indicated that the inhibition of STAT-3 completely abrogated the cardioprotection against ISO-induced injury by DG post-treatment in rats (unpublished data), implicating the involvement of STAT-3 activation in DG myocardial post-conditioning. Prior to an ischemic insult, treatment with puerarin (0.24 mmol/L in perfusate for 5 min) or daidzein (10 mg/kg, ip), both of which are ingredients in the DG extract, conferred cardioprotection against ischemia/reperfusion injury in rats both *in vitro *and *in vivo *by opening calcium-activated potassium channel and activating PKC or inhibiting nuclear factor-kappa B activation respectively [43-45]. Interestingly, intravenous administration of a mixture of puerarin and danshensu prior to an ischemic insult also protected against myocardial ischemia/reperfusion injury in rats through antioxidant actions [8].

## Conclusion

DG post-treatment protected the myocardium against ISO-induced acute injury in rats. The myocardial postconditioning by DG is likely mediated by signal pathway(s) inducing the activation of PKCε and mK_ATP_.

## Abbreviations

AST: aspartate aminotransferase; CHD: coronary heart disease; CPK: creatine phosphokinase; DG: Danshen-Gegen Decoction; GPX: selenium-glutathione peroxidase; GRD: glutathione reductase; GSH: reduced glutathione; ICDH: isocitrate dehydrogenase; ISO: isoproterenol; LDH: lactate dehydrogenase; MDA: malondialdehyde; mK_ATP_: mitochondrial ATP-sensitive potassium channel; MPT: mitochondrial permeability transition; PKCε: protein kinase C-epsilon; RISK: reperfusion injury salvage kinase; ROS: reactive oxygen species; SAFE: survivor activating factor enhancement; STAT-3: signal transducers and activators of transcription protein-3

## Competing interests

The authors declare that they have no competing interests.

## Authors' contributions

KMK designed the experiments. SMW, PYC and HYL performed the pharmacological experiments. LZ and ZZ performed the chemical analysis of the DG extract. SMW, PYL and KMK wrote the manuscript. All authors read and approved the final version of the manuscript.
